# Editorial: Alternative Therapeutic Approaches For Multidrug Resistant *Clostridium difficile*

**DOI:** 10.3389/fmicb.2019.01216

**Published:** 2019-05-31

**Authors:** Tavan Janvilisri, Joseph A. Sorg, Joy Scaria, Michael J. Sadowsky

**Affiliations:** ^1^Department of Biochemistry, Faculty of Science, Mahidol University, Bangkok, Thailand; ^2^Department of Biology, Texas A&M University, College Station, TX, United States; ^3^Department of Veterinary and Biomedical Sciences, College of Agriculture & Biological Sciences, South Dakota State University, Brookings, SD, United States; ^4^South Dakota Center for Biologics Research and Commercialization, Brookings, SD, United States; ^5^Biotechnology Institute, University of Minnesota, Saint Paul, MN, United States; ^6^Department of Soil Water and Climate, and Department of Plant and Microbial Biology, University of Minnesota, Saint Paul, MN, United States

**Keywords:** *Clostridium difficile*, therapeutics, multidrug resistance, alternative therapy, antibiotics

*Clostridium difficile* infection (CDI) is among the leading causes of infectious diarrhea among patients in hospitals and is increasing in the community. Treatment with antibiotics, especially those with a broad spectrum of activities, disrupt normal intestinal flora and create a dysbiotic state that favor acquisition and proliferation of *C. difficile*. Therefore, antibiotic use is the primary risk factor for the development of CDI among hospitalized patients. The first lines of treatment for CDI include metronidazole and vancomycin. However, an emergence of hypervirulent strains, which are characteristically resistant to such antibiotics, contributes to an increase in numbers of CDI cases worldwide. Resistance to antibiotics in *C. difficile* alleviates effective chemotherapy of CDI. Furthermore, CDI patients might be more likely to pick up an infection, as *C. difficile* becomes more resistant and/or could spread resistances to other bacteria. The clinical impact of resistance is therefore immense, characterized by increased cost, length of hospital stay, and mortality, posing a major cause for concern within healthcare and hospital environments. Consequently, there is an urgent need for alternative therapeutic approaches to treat drug resistant *C. difficile*.

This Research Topic “Alternative Therapeutic Approaches For Multidrug Resistant *Clostridium difficile*” emphasizes various strategies for combating antibiotic resistance in *C. difficile*, including novel antimicrobials from different sources such as oligopeptides, small molecules, and herbal medicine. Therapeutic alternatives are also presented and include phage therapy, fecal transplantation, and microbiota restoration. This Research Topic area comprises two reviews, one mini review, and seven original research articles, representing a broad spectrum of experimental approaches and areas of investigation to address alternative measures to tackle drug resistance in *C. difficile*.

There are attempts to identify novel compounds for the treatment of CDI. A study by Yang et al. revealed that lauric acid, a medium-chain fatty acid, exhibited an inhibitory effect on *C. difficile* vegetative cell growth, spore outgrowth, and biofilm formation. It has been shown that the cytotoxic effect of this compound was mediated via production of reactive oxygen species and plasma membrane damage. Using a mouse model of CDI, the pre-treatment with lauric acid could reduce inflammation caused by *C. difficile* toxin. Another study by Kers et al. demonstrated that variants of Mutacin 1140, a lantibiotic produced by the Gram- positive bacterium *Streptococcus mutans*, could serve as a backbone for novel antimicrobials against *C. difficile*. The promising OG253, the Phe_1_Ile variant of Mutacin 1140, has been shown to be potent against *C. difficile in vitro*, with low toxicity to mammalian cells and high stability. Furthermore, the treatment with OG253 could prevent CDI relapse in a hamster model. In another study by Harnvoravongchai et al. asiatic acid, a triterpenoid from a tropical plant *Centella asiatica* was assessed for its potential as an antimicrobial alternatives against *C. difficile*. The compound exhibited bactericidal activity, potentially through cell membrane damage, without interacting with vancomycin and metronidazole. It was also shown to possess negative effects on cell motility. Altogether, these compounds could be further developed as alternative treatments to combat CDI.

Recently, the U.S. FDA approved three new antimicrobial agents against Gram-positive bacteria—Tedizolid, Dalbavancin, and Ceftobiprole. Binyamin et al. attempted to evaluate the *in vitro* activity of these three antibiotics against 84 strains of *C. difficile* using the E-test method. They found that dalbavancin and tedizolid could be potential therapeutic agents for the treatment of CDI. Furthermore, dalbavancin, which inhibits cell wall synthesis, was superior compared to the first-line drug vancomycin, and the beta-lactam ceftobiprole exhibited lower MIC compared to the third generation beta-lactam ceftriaxone. In a quest to search for antibiotic alternatives, Thanissery et al. developed an *in vitro* screening pipeline to evaluate molecules as potential non-antibiotic therapeutics for CDI. They showed that 2-aminoimidazole molecules, could inhibit the growth and toxin activity of *C. difficile*, without disrupting normal gut flora, although this compound does not interfere with *C. difficile* sporulation.

Antibody-based immunotherapies are currently under development for the treatment of CDI. In an excellent review article, Péchiné et al. established that targeting *C. difficile* surface components represents alternative strategies to combat CDI. They provided an overview of characterized *C. difficile* surface components and the host specific immune response. Comparative views on passive immunization with various types of antigens are explored. A large number of potential vaccine strategies to prevent or cure CDI and recurrences have also been discussed. Another example of the immunotherapy against CDI is given in a mini review by Forster et al.. The authors summarized how antibody-mediated therapy could be applied for treatment and prevention of CDI. They explored antibodies in the clinical development stage, that are given systematically including Actoxumab and Bezlotoxumab as well as orally such as a bovine antibody from hyperimmune colostral milk with their perspective on the effective employment as non-antibiotic interventions.

Over the past decade, a number of non-antibiotic approaches for CDI treatment have been proposed. In an outstanding review, Baktash et al. discusses the mechanistic insights in the success of fecal microbiota transplants (FMT) for the treatment of CDI. The rationale of using FMT against *C. difficile* is discussed with its possible effects on *C. difficile* life cycles, including colonization resistance by healthy microbiota, suppression of *C. difficile* spore germination and outgrowth by modulating bile acids. Bacteriophages have also gained tremendous attention as promising antibiotic alternatives against resistant bacteria. Phothichaisri et al. made an effort to isolate and characterize phages specific to *C. difficile*. A total of 5 temperate phages belonging to the Myoviridae family were identified. The authors demonstrated that one of the phages targeted the S-layer protein of *C. difficile* cell wall. These phages could therefore lead to development of novel therapeutic agents and detection strategies for *C. difficile*. It is also well known that colonization resistance is an effective method to combat CDI. Vedantam et al. developed two synthetic biologic agents, Syn-LAB 2.0 and Syn-LAB 2.1, which were derived from lactic acid bacteria, with a host-cell binding fragment of the *C. difficile* adhesin SlpA on their cell-surface. They demonstrated that both biologics were safe and tolerable in hamster and piglet models with high colonization rate and exhibited protective effects against CDI in animals. Thus, these synthetic biologics could be of interest for investigators and clinicians as an alternative resource for tackling *C. difficile*.

At present, the problems concerning antibiotic resistance are evident, especially in the case of CDI, where treatment with antibiotics is a risk factor for the disease. It will be a challenge to search for alternative measures against CDI. The editorial team hopes that this Research Topic will be useful for investigators in the field. Finally, we would like to thank the authors for their contributions in this Research Topic, and all the reviewers for their critical review of the manuscripts.

## Author Contributions

All authors listed have made a substantial, direct and intellectual contribution to the work, and approved it for publication.

### Conflict of Interest Statement

The authors declare that the research was conducted in the absence of any commercial or financial relationships that could be construed as a potential conflict of interest.

